# A rule-based named-entity recognition method for knowledge extraction of evidence-based dietary recommendations

**DOI:** 10.1371/journal.pone.0179488

**Published:** 2017-06-23

**Authors:** Tome Eftimov, Barbara Koroušić Seljak, Peter Korošec

**Affiliations:** 1 Computer Systems Department, Jožef Stefan Institute, Ljubljana, Slovenia; 2 Jožef Stefan International Postgraduate School, Ljubljana, Slovenia; 3 Faculty of Mathematics, Natural Science and Information Technologies, Koper, Slovenia; University of Lisbon, PORTUGAL

## Abstract

Evidence-based dietary information represented as unstructured text is a crucial information that needs to be accessed in order to help dietitians follow the new knowledge arrives daily with newly published scientific reports. Different named-entity recognition (NER) methods have been introduced previously to extract useful information from the biomedical literature. They are focused on, for example extracting gene mentions, proteins mentions, relationships between genes and proteins, chemical concepts and relationships between drugs and diseases. In this paper, we present a novel NER method, called drNER, for knowledge extraction of evidence-based dietary information. To the best of our knowledge this is the first attempt at extracting dietary concepts. DrNER is a rule-based NER that consists of two phases. The first one involves the detection and determination of the entities mention, and the second one involves the selection and extraction of the entities. We evaluate the method by using text corpora from heterogeneous sources, including text from several scientifically validated web sites and text from scientific publications. Evaluation of the method showed that drNER gives good results and can be used for knowledge extraction of evidence-based dietary recommendations.

## Introduction

Nutritional sciences, such as clinical nutrition, food and nutrition management, public health nutrition, etc., combine a strong foundation in the biological, chemical and medical sciences, with a focus on nutrient/non-nutrient function and metabolism. The main objective of nutritional sciences is to establish food-based dietary guidelines (FBDGs) to achieve optimum health and the treatment or prevention of disease conditions as well as food production and safety [1]. Even though FBDGs are simple messages, we must be aware that they are based on complex scientific facts, which include dietary reference values (DRVs). DRVs are nutrient recommendations and quantitative reference values for nutritional intakes, such as population reference intake, the average requirement, adequate intake level, and the lower threshold intake. Authorities (e.g. European Food Safety Agency (EFSA) [2]) continuously identify and review the latest scientific studies, including reports of national and international authorities, for possible health effects of specific nutrients. For example, if the focus is on dietary fiber, health effects of dietary fiber are identified by reviewing scientific studies. Then, evidence of relationships between the intake of a nutrient and health outcome is evaluated. Finally, when nutrient-health relationships are established, the authority provides scientific advice that can be used by policy makers. In practice, this means that a daily intake of 25 g of a dietary fiber is set as a DRV because it is adequate for adults, while consuming greater than 25 g of dietary fiber per day may reduce the risk of coronary heart disease and type 2 diabetes and may improve weight maintenance [2]. Most countries have established their own national DRVs that consider, beside international recommendations and guidelines, also local conditions and national/ethnical eating culture and habits, and are reviewed and updated from time to time. A comprehensive review of micronutrient recommendations in Europe, collected within the EU-funded project EURRECA (EURopean micronutrient RECommendations Aligned) [3], was published several years ago in [4]. In 2015, the non-profit association EuroFIR [5] updated EURRECA micronutrient recommendations, enriched them with reference values for other nutrients, and developed a web service for accessing DRVs through the Quisper server platform [6], aimed at collecting scientifically-validated food-related data and knowledge services for dietary advising. Beside DRVs for the public, there also exist disease-specific DRVs aimed at increasing the awareness of clinicians and persons with chronic disease about beneficial nutritional therapies. Recently, personalized DRVs have become relevant as they consider genetic predisposition to chronic disease and phenotype information on anthropometry, physical activity, clinical parameters, and biochemical markers of nutritional status, and give strategies to dramatically reduce the risk of chronic-disease. The EU-funded Food4Me project [7] performed a pan-European study of over 1,500 participants, which showed that personalized advice is more effective at improving dietary behavior compared to conventional, population-based FBDGs [8].

In public health as well as in clinical practice, dietary recommendations should be based on evidence-based principles, considering scientific knowledge, expert consensus, and clinical experience.

Both DRVs and FBDGs are relevant and need to be combined in order to develop advanced health applications such as calculating nutritional values of dishes [9, 10], health recommendation systems [11–15], etc. The problem with these applications is that they require complete and the latest knowledge about DRVs and FBDGs. Another problem is that existing resources consist of a vast amount of both structured and unstructured data and information. Recent developments in ICT and Computer Science enables collection of the latest knowledge by exploiting the recently published biomedical literature and scientifically validated public health web sites. These resources lack coded data (e.g., unique identifiers from ontologies), but do have a lot of unstructured text that needs to be analyzed to correctly interpret dietary information. The amount of information presented as unstructured text is huge and is growing rapidly, computer-based tools for systematic knowledge identification, extraction, and exploration are welcome to support human experts when making decisions about appropriate nutritional care for specific disease states or conditions in typical settings.

There are several questions that need to be considered in order to extract relevant knowledge. Having the dietary information represented as unstructured text, the knowledge that needs to be extracted is related to DRVs with corresponding life stage groups, gender groups, and reference values for heights and weights for life stage and gender groups, and food composition data that usually contains information for a huge number of components, such as energy, macronutrients (e.g. protein, carbohydrate, fat), and their components (e.g. sugars, fatty acids), minerals (e.g. calcium, iron, sodium), and vitamins. So the first question is how to select parts of the text (phrases) that will be candidates for the entities in which we are interested. For this purpose, a good tokenization needs to be applied, such that each phrase can be a candidate for an entity and a phrase should not contain information about more than one entity. For example, let us have the recommendation “*The recommended intake for total fiber for adults 50 years and younger is set at 38 g for men and 25 g for women, while for men and women over 50 it is 30 g and 21 g per day, respectively, due to decreased food consumption.*” [16]. In this example, it is preferred to obtain “*the recommended intake for total fiber*” instead of “*fiber*” because it contains information about DRVs. Also, another question is how to extract all useful information from this recommendation and then to relate it together. The information that for “*adults 50 and younger*” the recommended intake is “*38 g for men*” and “*25 g for women*”, while the recommended intake for “*men and women over 50*” is “*30 g*” and “*21 g per day*”. Then, let us have another dietary recommendation, “*Some breakfast cereals contain 150 to 300 mg of sodium before milk is added.*”. In this example, the phrase “*150 to 300 mg of sodium*” is not preferred because it contains information about more than one entity. It is preferred to have two different phrases “*150 to 300 mg*” and “*sodium*”. Then, a good and representative knowledge base for each entity we are interested in needs to be selected, in order to link each phrase to find a set of candidates for each entity. An additional information is also reported in the action of the recommendation, such as “*contain*”, “*consist*”, “*should further increase*”, etc.

Automatic identification and classification of words or phrases that describe important concepts (entities) can be done by a process known as Named Entity Recognition (NER) [17]. NER is a process in which a label (class) or semantic category from a predefined set is assigned to the words or phrases known as entity mentions in order to describe the concept. There exist terminological-driven NER methods that aim to map mentions of concepts within texts to terminological resources, rule-based NER methods that use regular expressions of dictionary information with some characteristics of the entities of interest, and corpus-based NER methods that use evidence from text corpora and usually use machine learning (ML) approaches. Each of these different versions of NER method has its own advantages and limitations.

Different ML approaches have been applied in the extraction of concepts from the biomedical literature and the relations that exist between them. For example, there exist NER methods used for extracting relationships between genes and proteins, disease-phenotype relationships, chemical entities, and relationships between drugs and diseases, etc., but to the best of our knowledge there is no research that is focused on the extraction of dietary information concepts and on the relations that exist between them. Even more, for most machine learning approaches an annotated corpora in the domain is required. The annotated corpora in each domain is done by experts from the domain and it requires time and effort to produce it. In the domain of dietary recommendations an annotated corpora provided by the experts is still missing.

In this paper, we present a method that is an extension of our previous work [18], which was focused only on knowledge extraction of dietary information from a single sentence. We extended the method with several modifications. First, the extended version of the method works with text that could be a paragraph that contains more sentences instead of working only with one sentence. The sentence segmentation is introduced and each sentence is additionally split into more segments according to a set of rules in order to extract more useful information. Splitting the sentences into more segments improves the results that we obtained in our first attempt. New representative dictionaries, that are used for the entities we are interested in, were also added to improve the obtained results. For the evaluation of the method, instead of using a single sentence as one document as in our previous attempt, a test corpora was created, which contains documents form heterogeneous sources. We continue by explaining the related work, which includes the basic concepts for natural language processing (NLP) and ML, the definition of NER, and the overview of information extraction from the biomedical literature. Then, the newly proposed NER method for knowledge extraction of evidence-based dietary recommendations is explained in detail. At the end, the results and a discussion evaluating the proposed NER method are presented.

## Related work

In this section, we start by explaining the basic concepts of NLP and ML. Then, we give an explanation of the named entity recognition process that is used for automatic extraction of the useful information from the text. Finally, we give an overview of existing problems and solutions for information extraction from the biomedical literature.

### Natural language processing and machine learning

NLP is a research area of computer science, artificial intelligence, and computational linguistics, concerned with the interactions between computers and human natural languages. More information about NLP can be found in [19, 20]. NLP works with data represented as unstructured text, which depends on how people express themselves. Text is processed by sentence segmentation and further the segments are analyzed by applying tokenization that is a process of breaking the segments into words that are called tokens. Each of the tokens consists of a string of characters without white space. The tokens can be analyzed by applying lemmatization [21] or stemming [22, 23]. From linguistics, the lemmatization is the process of grouping together different inflected forms of a word so they can be analyzed as a single item. The uninflected form is called lemma. In computational linguistics, the lemmatization is a process of determining the lemma of a given token (word). It usually works by using vocabulary and morphological analysis of the token in order to return the lemma or dictionary form of the token. Stemming is another approach similar to lemmatization. It usually works by removing the suffixes of the token in order to give a good approximation to the lemma. Further, the tokens can be analyzed by applying part-of-speech (POS) tagging that is a process of assigning morphological tags or categories (classes) to each token (e.g, NN (noun, singular or mass), VB (verb, base form), and VBD (verb, past tense)) [24–28]. Sometimes it can happen that we are not interested into tokens, but we want to determinate text phrases that are concepts (entities). Chunking [20] is one approach that uses POS tags and identifies short phrases such as noun phrase (NP), verb phrase (VP), preposition phrase (PP), etc. Chunking is usually combined with B-I-O tagging scheme, which gives a tag to each token at the beginning of the phrase (B), inside the phrase (I), and outside of any phrase that is tagged (O). For example, the noun phrase (B-NP, I-NP) consists of two tokens, in which the first token is the beginning of the noun phrase and the second token is inside the noun phrase. Despite the morphological analysis, the sentences can be analyzed according to their syntactic structure. The process of working with the syntactic analysis of the sentences is called parsing [29].

Alternatively, we have ML, which is a subfield of computer science related to studies of pattern recognition and computational learning theory in artificial intelligence [30]. It focuses on developing algorithms that can learn and make predictions based on data. The data is presented as a training set, which is a collection of instances described by attributes called features. If the training set consists of output labels (classes), given by an expert from the data domain, that are the desired output of the algorithm, then we have supervised learning. If the desired output labels are not present in the training set, we have unsupervised learning, the goal of which is to find some hidden patterns in the data. Also, semi-supervised learning exists, which is a combination of supervised and unsupervised learning. The idea of the ML algorithms is to give further analyses of new unseen instances that are not present in the training set. For example, in supervised learning the output label (class) of these new unseen instances needs to be predicted using the algorithm. Because the ML supervised algorithms perform well for the instances from the training set, their evaluation needs to be done by using a test set that consists of instances that are not found in the training set. For this purpose, the training set is often randomly split into two portions, the training set and the test set. Another approach for evaluating the performance of ML supervised algorithms is to use cross-validation [31].

ML supervised algorithms are the most used algorithms for information extraction from text. They are based on annotated corpora, which include text in which the labels of the entities of interest are assigned by domain experts. Using it, different ML models such as decision trees [32], support vector machines (SVMs) [33], hidden Markov models [34], conditional random fields (CRFs) [35], maximum entropy [36], etc., can be applied in order to achieve better performance. Moreover, the idea of ensemble learning [37] can be used to combine multiple learning algorithms to obtain better performance that could be obtained from any of the constituent learning algorithms alone.

### Named entity recognition

Named entity recognition (NER) [17] is a part of information extraction that aims to determine and identify words or phrases in text into predefined labels (classes) that describe concepts of interest in a given domain. There exist various NER methods.

Terminology-driven NER methods, also called dictionary-based NER methods [38–40] work by matching the text phrases with concept synonyms that exist in the terminological resources (dictionaries). In order to improve the performance of these methods, instead of strict matching they are combined with some heuristics such as the generating of words that occur in entity mentions, generating permutations of words in concept synonyms, solving disambiguation problem, etc. The main disadvantage of these methods is that only the entity mentions that exist in the resources will be recognized, but the benefit of using them is related to the frequent updates of the terminological resources with new concepts and synonyms.

Another NER methods are rule-based NER methods [41, 42], which use regular expressions that combine information from terminological resources and characteristics of the entities of interest. The main disadvantage of these methods is the manual construction of the rules, which is a time-consuming task and depends on the domain.

Corpus-based NER methods [43–46] are based on the evidence that exists in an annotated corpora provided by human experts from the domain and use of ML algorithms to predict the entities labels. These methods are less affected by terminological resources and manual created rules, but the limitation is the existence of an annotated corpora for the domain of interest. The construction of the annotated corpora for a new domain is a time consuming task and requires effort by the human experts to produce it.

### Overview of information extraction from biomedical literature

Nowadays, the information extraction from the biomedical literature is a very important task in order to improve public health. Because the NER methods with best performances are usually corpus-based NER methods, there is a need for annotated corpora from biomedical literature that will include the entities of interest. For this purpose, different annotated corpora are produced by shared tasks, where the main aim is to challenge and encourage research teams on NLP problems.

BioNLP Shared Task 2013 [47] aims to provide a common framework for information extraction in the biomedical domain. The biological questions addressed by this task were related to the molecular biology domain and its related fields. The BioNLP Shared task 2013 consists of six tasks: gene event extraction, cancer genetics, pathway curation, corpus annotation with gene regulation ontology, gene regulation networks in bacteria, and bacteria biotopes. BioNLP Shared Task 2016 comprises three tasks that address different aspects of knowledge acquisition from text and also encompasses a wide range of biological diversity [48–56]. The SeeDev task [48] aimed at extracting the regulation of the seed development in plants using a rich model. The Bacteria Biotopes 3 (BB3) task [52, 54] was used for the construction of a bacteria habitat database using external ontologies. The Genia 4 (GE4) task [50] aimed at delivering a new shared task framework to construct a knowledge base of NFkB synthesis and regulation through IE.

BioCreative II gene mention recognition [57] was a task, where different systems were designed to identify substrings in sentences corresponding to gene name mentions. The annotated corpora was provided to the participants, on which different methods were used and results in the performances varied. The best system was a semi-supervised learning method known as alternating structure optimization (ASO) [58]. Other systems were developed by using supervised ML algorithms. The second best performing system used CRFs [59], the third best performing system used a combination of two SVMs and one CRF [60], and the fourth best performing system used a multimodal approach with two CRFs [61]. For example, one system that is evaluated on the BioCreative 2 GM task training corpus is BANNER [62]. This is an open-source biomedical named-entity recognition system implemented using CRF. It represents an innovative combination go known advances beyond the existing open-source systems, in a consistent, scalable package that can easily be configured and extended with additional techniques. The work on gene mention recognition continued in BioCreative III [63], where the focus was on three tasks: cross-species gene normalization using full text, extraction of protein-protein interactions from full text, including document selection, identification of interacting proteins and identification of interacting protein pairs, and an interactive demonstration task for gene indexing and retrieval task using full text. In BioCreative IV [64], the gene ontology annotation task was reintroduced along with the following new tasks: interoperability of text mining systems, web service-based named entity recognition, and chemical/drug entity name recognition. In the chemical/drug named entity recognition two main aspects were covered, the chemical document indexing and the chemical entity mention recognition. The extraction of chemical entities from unstructured text is a very important task for different research areas, because they are related with metabolism, enzymatic reactions, potential adverse effects, etc. The systems presented are based on three general strategies: supervised ML approaches, rule/knowledge-based approaches, and chemical dictionary look-up approaches [65]. The evaluation of the systems performances was made using the CHEMDNER annotated corpus that was provided by the workshop [66]. Most systems that use supervised ML methods are based on the CRFs, some of them used SVMs, and some a combination of SVMs and CRFs. There were presented systems that use mainly rule-based methods, but these require a deep understanding of both the existing chemical nomenclature standards as well as of the CHEMDNER annotation guidelines. The use of dictionary-lookup based systems required efficient dictionary pruning and post-processing of the results. For example, becas [67] provides annotations for isolated, nested, and intersected entities. It uses dictionary-matching techniques to recognise species, anatomical concepts, microRNAs, enzymes, chemicals, drugs, diseases, metabolic pathways, cellular components, biological processes, and molecular functions. It gives an opportunity to choose the types of entities in which you are interested. It was validated against CRAFT [68], AnEM [69], and NCBI Diseases corpora [70], achieving an f-measure of 76% for genes and proteins, 95% for species, 65% for chemicals, 83% for cellular component, 92% for cells, 63% for molecular functions and biological processes, 83% for anatomical entities, and 85% for diseases. Becas[chemicals] [71] is a web application and API for recognizing and annotating chemical compounds and drugs. It is a special branch of becas API focusing on identifying of a large array of chemical substances. It uses machine-learning techniques, with an optimized feature set including orthographic, morphological, natural language processing, domain knowledge, and local context features. It was validated against the BioCreative IV CHEMDNER task corpora, achieving an f-measure of 87.48% for chemical named entities. The work related to chemical named entity recognition continued also in BioCreative V [72–74], where the focus was on disease and symptoms related entities and relations that exist between chemical/drug entities and disease entities. BioCreative 2016 was focused on four main tasks: applications of text mining methods in areas such as crowdsourcing, database curation, the publication process, and metagenomics; methods for annotations such as disease, phenotype, and adverse reactions in different text sources literature, clinical records and social media; methods to achieve interoperability, generalisability, and scalability in text mining: BioC [75], RDF and semantic web, among others; and the application of ontologies in text mining and text mining as an ontology builder.

From an overview of the existing information extraction methods from the biomedical literature, we can see that a lot of NER methods exist in the domain of biomedical literature and they are focused on different biomedical domains. The commonly used NER methods are the corpus-based NER methods that rely on annotated corpora for the domain of interest, which is produced by the domain experts. We did not find any research that focuses on extracting dietary information from text. Also, we could not find an annotated corpora that can be used to developed corpus-based NER methods for dietary information. Therefore, we have developed a new NER method for knowledge extraction of evidence-based dietary recommendations from unstructured text data that is not annotated. The method uses a combination of NLP techniques with Boolean algebra rules and matrix theory in order to extract the entities from the domain.

## Methods

### A rule-based NER method for information extraction of evidence-based dietary recommendations

The dietary information presented in the evidence-based dietary recommendations is provided through the information about DRVs and FBDGs. Because an annotated corpora in this domain is still missing, after several discussions with the human experts from the domain, we realized that the basic dietary information that needs to be extracted is represented with food entities, *Food*, nutrient entities (or chemical components), *Nutrient*, and associated quantity/unit entities, *Quantity*/*Unit*. Also, there is a need to track an additional information that is reported as an action in the recommendation (e.g, increase, decrease, contains, etc.) in order to properly interpret the information provided. Despite the main entities that are important, the recommendation can also consist of other useful information that needs to be tracked such as life stage group of the population, national characteristics of the population, cultural habits, etc.

In order to extract the dietary information provided by evidence-based dietary recommendations, we proposed a novel rule-based NER method, that we call drNER. It is a combination of the terminological-driven NER and rule-based NER. The difference with the purely terminological-driven NERs is that we do not use only dictionaries with concepts and synonyms, but we allow the reuse of some corpus-based NERs that exist for some entities. So if corpus-based NERs exist for some entities we are interested in, we can use these systems to annotate the text data and then to see if some tokens have labels that correspond to entities of interest. We also combine corpus-based NERs that exist for some entities in which we are interested, following the idea of ensemble learning in order to achieve better performance that could be obtained from any corpus-based NER alone. The difference with the rule-based NERs is that we do not use rules associated with the characteristics of the entities. This is because having rules for each of the entities we are interested in, requires too much time and effort to produce them. We only used a small number of Boolean algebra rules that are not related to the characteristics of the entities, but help us define the phrases that are entities mentions.

**Fig 1:**
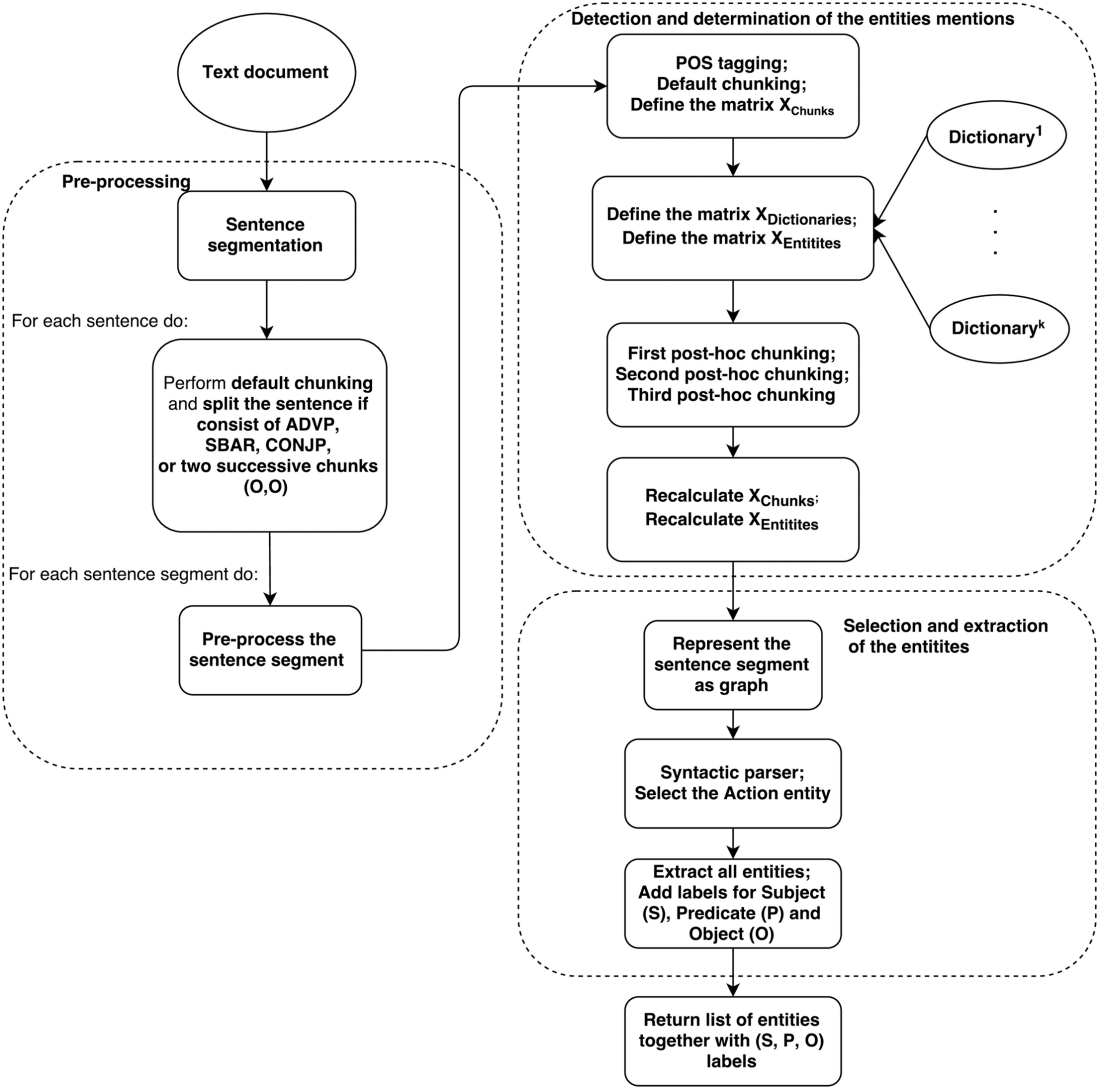
DrNER workflow diagram.

DrNER works with text that is composed of sentences and paragraphs. First, we split the text using sentence segmentation. Then, each sentence is split with an additional segmentation and each sentence segment is pre-processed and analyzed in two phases. The first one is detection and determination of the entities mentions. It is based on a combination of NLP methods, matrix theory, and boolean algebra. The second one is the selection and extraction of the entities. It uses the information from the first phase by representing it using graph theory and a small set of rules that define how to extract the useful entities. The workflow diagram of the NER method is presented in Fig 1.

For a better understanding of the drNER, the pseudocode of the method is presented in Algorithm 1.

**Algorithm 1** drNER

1:    Apply sentence segmentation on the text document

2:    **for** each sentence, Φ_*i*_, in the set of sentences, Φ **do**

3:     Obtain *Chunks* vector by introducing the default chunking on Φ_*i*_

4:     Split Φ_*i*_ on the position of each *ADV P*, *CONJP*, *SBAR* chunk, or two successive chunks (*O*,*O*), into set Ψ

5:     **for** each sentence segment, Ψ_*i*_, in Ψ **do**

6:      Obtain the *Tokens* vector by using the word-level tokenization on Ψ_*i*_

7:      Obtain the *Chunks* vector by introducing the default chunking on Ψ_*i*_

8:      Obtain the *X*_Chunks_ matrix using the Eq (1)

9:      Obtain the *X*_Dictionaries_ matrix using the Eq (2)

10:   Obtain the *X*_Entities_ matrix using the Eq (3)

11:   Perform the first post-hoc chunking

12:   Perform the second post-hoc chunking

13:   Perform the third post-hoc chunking

14:   Recalculate the *X*_Chunks_ matrix using the Eq (1)

15:   Recalculate the *X*_Entities_ matrix using the Eq (3)

16:   Select the *Action* entity by searching the predicate in the VP subtrees

17:   Extract all entities

18:   Add the labels for (Subject, Predicate, Object) using *S*, *P*, *O*

19:  **end for**

20: **end for**

21: Return list of entities together with (Subject, Predicate, Object) labels

### Dictionaries

We start by explaining the dictionaries or the terminological resources we used for the drNER.

For the *Quantity*/*Unit* entity, an ontology, called Units of Measurements Ontology (UO) [76–78], is used. The UO is currently being used in many scientific resources for the standardized description of measurement units. From it, the names of the units together with their symbols are extracted. In addition, a list of measurement units that are used for recipes, such as tablespoon, teaspoon, etc. are added.

For the *Nutrient* and the *Food* entity, dictionaries are constructed using the outputs of different NERs appropriate for the entity.

For the *Nutrient* entity, a combination of becas API [67], becas[chemicals] API [71] and a semantic tagger, known as USAS online English semantic tagger [79–81], is used. Both, becas and becas[chemicals], are web-services-based and corpus-based NER developed as a prat of BioCreative IV CHEMDNER task. Becas provides annotations for isolated, nested, and intersected entities. It uses dictionary-matching techniques to recognise species, anatomical concepts, microRNAs, enzymes, chemicals, drugs, diseases, metabolic pathways, cellular components, biological processes, and molecular functions. It also gives an opportunity to choose the types of entities. Becas[chemicals] is a web application and API for recognizing and annotating of chemical compounds and drugs. It is a special branch of becas API focused on the identification of a large array of chemical substances. It uses machine-learning techniques, with an optimized feature set including orthographic, morphological, natural language processing, domain knowledge, and local context features. The USAS online English semantic tagger is a part of the UCREL semantic analysis system, which is a framework of automatic semantic analysis of text that has been designed across a number of research projects since 1990. The USAS version contains 21 major entity labels, with the possibility of subdivision for some of them. For our purpose, the focus is on one category with the semantic label *O*, which is for terms related to substances, materials, objects, and equipment. From this entity label, we use only one subcategory *O*_1_ that is for terms relating to substances and materials generally. The idea of using a combination of NER systems comes from the idea of ensemble learning in order to achieve a better performance of identifying the chemical entities that could be obtained from any of them alone. So if a token is annotated by at least one of these systems, as a chemical entity using becas and becas[chemicals] or *O*_1_ using the USAS tagger, then we can assume that the token belongs to this dictionary.

For the *Food* entity the same semantic tagger as the *Nutrient* entity, known as USAS online English semantic tagger, is used. The focus is on two categories. The first one is the category for terms related to Food and Farming, *F*. From it, four subcategories are used. The first subcategory is for terms related to food and food preparation, *F*_1_, the second is for terms related to drinks and drinking, *F*_2_, the third is for terms related to cigarettes and drugs, *F*_3_, and the forth for terms related to agriculture and horticulture, *F*_4_. The second category is for terms related to Life and Living things, *L*. From it, two subcategories are used. The first one is for terms related to living creatures (e.g. non-human), *L*_2_, and the second subcategory is for terms related to plants and plant-life, *L*_3_. In Table 1, the summary of the USAS English tagger categories and subcategories is presented. So if a token is annotated by this system as *F*_1_, *F*_2_, *F*_3_, *F*_4_, *L*_2_, or *L*_3_, then we can assume that the token belongs to this dictionary.

**Table 1:** USAS categories.

Entity	USAS category	USAS subcategory
Nutrient entity	**O**—Substances, materials, objects, and equipment	**O_1_**—Substances and materials
Food entity	**F**—Food and farming	**F_1_**—Food and food preparation
		**F_2_**—Drinks and drinking
		**F_3_**—Cigarettes and drugs
		**F_4_**—Agriculture and horticulture
	**L**—Life and living things	**L_2_**—Living creatures (e.g. non-human)
		**L_3_**—Plants and plant life

It is not possible to provide information about the size of various dictionaries being used because they are not classical dictionaries that consist of concepts with synonyms. For the *Quantity*/*Unit* entity, an ontology is used together with kitchen-related units. For the *Nutrient* entity, three corpus-based NER systems are used, so the results provided as annotations from these three systems are used and combined. Also, for the *Food* entity, a corpus-based NER system is used, so the results provided as annotations are used.

### Pre-processing

Before we start with knowledge extraction, the first step is to pre-process the text data. First, sentence segmentation is used for each text document. Then, default chunking [82] is introduced on each sentence. In our implementation, the Apache OpenNLP Maxent sentence detector is used for sentence segmentation and the Apache OpenNLP Maxent chunker is used for default chunking. They are part of the *openNLP* R package [82]. After default chunking, if the sentence consist of *ADV P*, *CONJP*, *SBAR* chunks, or two successive chunk tokens that are (*O*,*O*), we need to split the sentence on that place or places. The *ADV P* chunk is for an adverbial phrase, the *CONJP* is for a conjunctive phrase, the *SBAR* is for a subordinated clause, and (*O*,*O*) means that two successive tokens are outside of any chunk. Splitting the sentence into more segments is useful to extract more information that can stay hidden if the sentence is not split. Then for each sentence segment the double quotation marks and brackets are removed.

To explain the difference between sentence and sentence segments, we continue with an example. Let one sentence, obtained from sentence segmentation of a text document, be “*The recommended intake for total fiber for adults 50 years and younger is set at 38 g for men and 25 g for women, while for men and women over 50 it is 30 g and 21 g per day, respectively, due to decreased food consumptions.*” [16]. The result of the default chunking on this sentence is presented in Table 2. The column *Tokens* corresponds to the tokens obtained by the word-level tokenization and the column *Chunk tokens* corresponds to the chunk token obtained by the default chunking. Further, because this sentence consists of two *ADV P* chunks, it needs to be split on that places. After splitting, the obtained sentence segments are: “*The recommended intake for total fiber for adults 50 years and younger is set at 38 g for men and 25 g for women.*”, “*For men and women over 50 it is 30 g and 21 g per day.*”, and “*Due to decreased food consumptions.*”. These sentence segments are further used by the proposed method.

**Table 2:** Default chunking result for one sentence.

Token id	Tokens	Chunk tokens	Token id	Tokens	Chunk tokens
1	The	B-NP	26	while	**B-ADVP**
2	recommended	I-NP	27	for	B-PP
3	intake	I-NP	28	men	B-NP
4	for	B-PP	29	and	I-NP
5	total	B-NP	30	women	I-NP
6	fiber	I-NP	31	over	B-PP
7	for	B-PP	32	50	B-NP
8	adults	B-NP	33	it	B-NP
9	50	B-NP	34	is	B-VP
10	years	I-NP	35	30	B-NP
11	and	O	36	g	I-NP
12	younger	B-NP	37	and	O
13	is	B-VP	38	21	B-NP
14	set	I-VP	39	g	I-NP
15	at	B-PP	40	per	B-PP
16	38	B-NP	41	day	B-NP
17	g	I-NP	42	,	O
18	for	B-PP	43	respectively	**B-ADVP**
19	men	B-NP	44	,	O
20	and	O	45	due	B-PP
21	25	B-NP	46	to	I-PP
22	g	I-NP	47	decreased	B-NP
23	for	B-PP	48	food	I-NP
24	women	B-NP	49	consumptions	I-NP
25	,	O	50	.	O

### First phase: Detection and determination of the entities mentions

Let Φ be a sentence or sentence segment that contains dietary information. We start by introducing the word-level tokenization on Φ. The result is a *n* × 1 vector, *Tokens*, whose elements are the tokens from Φ, and *n* is the number of tokens obtained after the tokenization. Then we continue with POS (part-of-speech) tagging and the result is an *n* × 1 vector, *POS*_Tags_, which is a collection of POS tags for Φ.

After processing the sentence at the word-level, we continue with default chunking, which segments and labels multitoken sequences called chunks. The result is an *n* × 1 vector, *Chunks*, whose elements are chunk tokens tagged in the B-I-O tagging format.

The next step is to define an *n* × *m* matrix, *X*_Chunks_, where *m* is the number of chunk tokens from the *Chunks* vector that begin with the prefix B- or O. The elements of the matrix *X*_Chunks_ are defined by the Eq (1), so if the token belongs to the chunk we have 1, and 0 otherwise.

$$\begin{array}{c} X_{\text{Chunks}}\left[i,j\right]=\{\begin{array}{cc} 1, & \text{if}\;Tokens[i]\in Chunks[j]\\ 0, & otherwise \end{array}, \end{array}$$ (1)

where *i* = 1, ‥, *n* and *j* = 1, ‥, *m*.

Let *k* be the number of entities we are interested in, which in our case is 3, *Food*, *Nutrient*, and *Quantity*/*Unit*. In order to detect and determinate the entities mentions, we try to link each chunk with the information from additional terminological resources (dictionaries) related to the domains of the entities, *Dictionary*^*l*^, *l* = 1, ‥, *k*. Once more we would like to point out that it is not necessary that these dictionaries are standard resources, which consist of concepts with synonyms, but also they can be NER systems that exist for some entities from the domain, or even more a combination of NERs in order to achieve better performance. Then an *n* × *k* matrix, *X*_Dictionaries_, is defined as

$$\begin{array}{c} X_{\text{Dictionaries}}\left[i,l\right]=\{\begin{array}{cc} 1, & \text{if}\;Tokens\left[i\right]\in Dictionary^{l}\\ 0, & otherwise \end{array}. \end{array}$$ (2)

After obtaining the matrices *X*_Chunks_ and *X*_Dictionaries_, an *m* × *k* matrix, *X*_Entities_, is defined as

$$\begin{array}{c} X_{\text{Entities}}=X_{\text{Chunks}}^{T}\cdot X_{\text{Dictionaries}}. \end{array}$$ (3)

The rows of the matrix *X*_Entities_ correspond to the chunks and the columns are the dictionaries we included for the entities. For example if the element *X*_Entities_[*i*, *l*] ≥ 1, this means that the *i*-th chunk is an entity mention solution for the *l*-th entity. The additional step is to check if a chunk is an entity mention of more entities. If this is the case, then the chunk obtains the entity tag from the last token.

In most cases, the potential entity mentions are noun phrases that are the linguistically meaningful units, but sometimes it can happen that the entity mention can consist of more noun phrases or even combinations of noun phrases with some other morphological phrases. To improve the quality of text phrases that can be entities mentions, three additional post-hoc chunkings are introduced. The first post-hoc chunking combines the information from the default chunking and the entities labels for each chunk by defining Boolean algebra rules. The second post-hoc chunking uses the information from the first post-hoc chunking and the POS tags of the tokens. The last one combines the information from the second post-hoc chunking and the entities labels for each chunk by defining rules.

#### First post-hoc chunking

**Fig 2:**
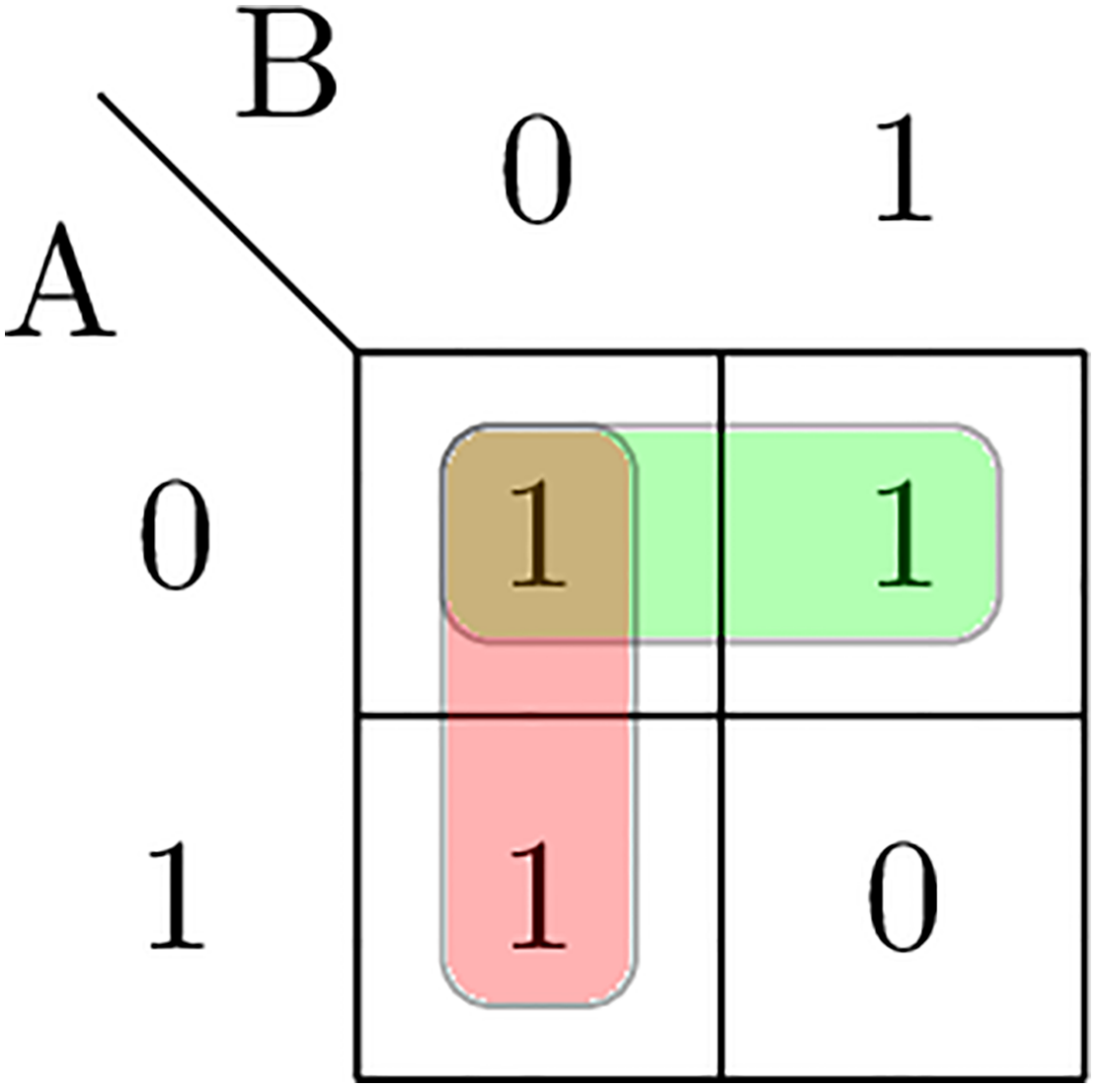
Karnaugh map of the Boolean function for the first post-hoc chunking.

In the first post-hoc chunking, trigrams of successive chunks (*Chunk*_*i*_, *Chunk*_*i*+1_, *Chunk*_*i*+2_) are analyzed and merged into one new noun chunk if the trigram is composed as (*B* − *NP*, *B* − *PP*, *B* − *NP*). This is done except in cases when the two noun chunks correspond to entity mentions of different entities because merging them can lose information about one entity described by one of the noun chunks. In order not to lose this information, we define a boolean function when this chunking needs to be performed. In Table 3 the boolean function, together with the boolean variables *A* and *B*, that in our case can be different entities, is presented. Further, a Karnaugh map Fig 2, also known as a K-map [83], is used to simplify the boolean algebra expression when this chunking needs to be performed. The boolean algebra expression or the boolean function, is obtained in a simplified form, as a sum of minterms, as

$$\begin{array}{c} f(A,B)=\neg A\vee \neg B=\neg (A\wedge B). \end{array}$$ (4)

Because the number of the entities we are interested in can be greater than 2, *k* > 2, the boolean algebra expression obtained using Eq (4) needs to be defined for each variation of pairs of entities. The number of functions is determined using the formula of the variations without repetition $V_{r,w}=\frac{r!}{(r-w)!}$, where *r* is the number of different elements, in our case the number of different entities, *k*, and *w* is the size of the variation or how many elements need to be selected from the set of *r* elements. In our case, *w* is 2 because we are working with a pair of entities. Then all the obtained functions are merged together with boolean *AND* conditions into one expression. This expression defines whether the first chunking needs to be performed.

**Table 3:** The Boolean function for the first post-hoc chunking.

A	B	f
0	0	1
0	1	1
1	0	1
1	1	0

To see how the first post-hoc chunking works, let us focus on one example using the sentence “*People of any age who are African Americans should further reduce sodium intake to 300 mg per day.*” [16]. This sentence does not consist of *ADV P*, *CONJP*, *SBAR* chunks, or two successive chunks that are (*O*,*O*), so it is not split into sentence segments. The results of the default chunking and linking each token to a dictionary is presented in Table 4. The *Tokens* column corresponds to the result of the word-level tokenization. The *POS tags* column corresponds to the result of the POS tagging. The *Chunk tokens* column corresponds to the result of the default chunking, where each chunk token is presented in B-I-O tagging format and the beginning of each new chunk is marked with the symbol *. The *Food*, *Nutrient*, and *Quantity/Unit* columns correspond to the linking of each token to the dictionaries used for each entity. In the column *Chunk_1_ tokens* the result of the first post-hoc chunking is presented, here only the new chunks formed are presented in bold font. From this table, we can see how each chunk consists of one or more chunk tokens. For example, “*sodium intake*” is one noun chunk that consists of two chunk tokens (*B* − *NP*, *I* − *NP*). After using the information given in this table and calculating the matrices *X*_Chunks_ and *X*_Dictionaries_, the matrix *X*_Entities_ is presented in Table 5.

**Table 4:** Example of first post-hoc chunking.

Tokens	POS tags	Chunk tokens	Food	Nutrient	Quantity/Unit	Chunk_1_ tokens
People	NNS	B-NP*	0	0	0	B-NP*
of	IN	B-PP*	0	0	0	**I-NP**
any	DT	B-NP*	0	0	0	**I-NP**
age	NN	I-NP	0	0	0	**I-NP**
who	WP	B-NP*	0	0	0	B-NP*
are	VBP	B-VP*	0	0	0	B-VP*
African	JJ	B-NP*	0	0	0	B-NP*
Americans	NNP	I-NP	0	0	0	I-NP
should	MD	B-VP*	0	0	0	B-VP*
further	RBR	I-VP	0	0	0	I-VP
reduce	VB	I-VP	0	0	0	I-VP
sodium	NN	B-NP*	0	**1**	0	B-NP*
intake	NN	I-NP	0	0	0	I-NP
to	TO	B-PP*	0	0	0	B-PP*
300	CD	B-NP*	0	0	0	B-NP*
mg	NN	I-NP	0	0	**1**	**I-NP**
per	IN	B-PP*	0	0	0	**I-NP**
day	NN	B-NP*	0	0	0	**I-NP**
.	.	O	0	0	0	O

^1^ indicates the result of the first post-hoc chunking

* indicates a beginning of new chunk

**Table 5:** X_Entities_.

Chunks	Chunk tokens	Food	Nutrient	Quantity/Unit
People	B-NP*	0	0	0
of	B-PP*	0	0	0
any age	B-NP*	0	0	0
who	B-NP*	0	0	0
are	B-VP*	0	0	0
African Americans	B-NP*	0	0	0
should further reduce	B-VP*	0	0	0
sodium intake	B-NP*	0	**1**	0
to	B-PP*	0	0	0
300 mg	B-NP*	0	0	**1**
per	B-PP*	0	0	0
day	B-NP*	0	0	0
.	O	0	0	0

* indicates a beginning of new chunk

Further, the information from the matrix *X*_Entities_ is used for the first post-hoc chunking. In order not to lose information by applying it, the boolean function, must be defined. In our case, we are interested in three entities, *Food*, *Nutrient*, and *Quantity/Unit*. By using the Eq (4), the function is defined for all variations of pairs of entities. In our case, the number of variations is *V*_3,2_ = 6. Let the trigram of successive chunks be (*Chunk*_*i*_, *Chunk*_*i*+1_, *Chunk*_*i*+2_). Then, the boolean function is defined as

$$\begin{array}{cc} & \neg \left(Nutrient_{i}\wedge Quantity/Unit_{i+2}\right)\wedge \neg \left(Quantity/Unit_{i}\wedge Nutrient_{i+2}\right)\\ & \wedge \neg \left(Nutrient_{i}\wedge Food_{i+2}\right)\wedge \neg \left(Food_{i}\wedge Nutrient_{i+2}\right)\\ & \wedge \neg \left(Food_{i}\wedge Quantity/Unit_{i+2}\right)\wedge \neg \left(Quantity/Unit_{i}\wedge Food_{i+2}\right), \end{array}$$ (5)

which is true only if there is no pair for which both values are one. In the example, there are three trigrams of successive chunks that satisfy the condition (*B* − *NP*, *B* − *PP*, *B* − *NP*). They are (“*People*”, “*of*”, “*any age*”), (“*sodium intake*”, “*to*”, “*300 mg*”), and (“*300 mg*”, “*per*”, “*day*”). For the first one, (“*People*”, “*of*”, “*any age*”), the boolean function defined by Eq (5) is true because all entity labels are 0. So this post-hoc chunking needs to be performed and the chunks are merged into one new noun chunk, “*People of any age*”. For the trigram (“*sodium intake*”, “*to*”, “*300 mg*”), the boolean function is false because the label *Nutrient*_*i*_ = 1 and the label *Quantity*/*Unit*_*i*+2_ = 1, so there is a pair where both values are one. According to this, ¬(*Nutrient*_*i*_ ∧ *Quantity*/*Unit*_*i*+2_) = 0 and the value of the boolean function is false, so in this case the first post-hoc chunking should not be performed. In this example, if we merged the chunks we will lose information about one entity. For the last trigram (“*300 mg*”, “*per*”, “*day*”), the label *Quantity*/*Unit*_*i*_ = 1, while other labels are *Nutrient*_*i*+2_ = 0, *Food*_*i*+2_ = 0, so the boolean function is true. In this case, the first post-hoc chunking should be performed and the new chunk is “*300 mg per day*”.

#### Second post-hoc chunking

In the second post-hoc chunking, trigrams of successive chunks obtained by the first post-hoc chunking (*Chunk*_*i*_, *Chunk*_*i*+1_, *Chunk*_*i*+2_) are analyzed and merged into one new noun chunk if the trigram is composed as (*B* − *NP*, *B* − *V P*, *B* − *NP*) and the first noun chunk has a POS tag that is a Wh-pronoun [27], such as who, what, which, etc.

To see how the second post-hoc chunking works, the obtained result from the example of the first post-hoc chunking is used. In this post-hoc chunking we are interested in the trigrams of successive chunks that satisfy the condition (*B* − *NP*, *B* − *V P*, *B* − *NP*). By using the obtained result from the first post-hoc chunking, which is presented in Table 5, column *Chunk_1_ tokens*, there are two trigrams that satisfied this condition, (“*who*”, “*are*”, “*African Americans*”) and (“*African Americans*”, “*should further reduce*”, “*sodium intake*”). From them, only the first one has the first noun chunk that is a Wh-pronoun, so they are merged together, “*who are African Americans*”.

#### Third post-hoc chunking

In the third post-hoc chunking, bigrams of successive chunks obtained by the second post-hoc chunking (*Chunk*_*i*_, *Chunk*_*i*+1_) are merged into one new noun chunk if the bigram is composed as (*B* − *NP*, *B* − *NP*) and only one of the noun chunks is labeled as an entity of interest, or both of them have the same label.

By using the obtained result from the example of the second post-hoc chunking, there are no bigrams of chunks that satisfied the condition (*B* − *NP*, *B* − *NP*), and only one of the noun chunks is labeled as an entity of interest, or both of them have the same label.

After performing the three post-hoc chunkings, the matrix *X*_Chunks_ needs to be recalculated because the number of chunks is different from the number obtained by the default chunking. At the end of the first phase, the matrix *X*_Entities_ is recalculated and their columns correspond to the sets of entity mentions for each entity.

### Second phase: Selection and extraction of the entities

The result from the first phase of the NER method are sets of entities mentions for each entity. The next step is the second phase in which the entities mentions form the sets that contribute to the dietary information need to be selected.

For this purpose, the sentence or sentence segment is represented as a graph, in which each chunk is connected only with its neighbors (predecessor and successor chunk). Then the start or initial node of the graph from where the search for all entities begins, is selected using syntactic bracketing or tree parsing. Each sentence or sentence segment, Φ, is represented by the parser as a tree having three children: a noun phrase (NP), a verbal phrase (VP) and a full stop (.). In addition, each sentence is formed as a combination of three parts, *Subject*, *Predicate*, and *Object* [84]. The *Subject* is the person or a thing who or which carries out the action of the verb. The *Predicate* in a sentence is what us tells about what a person or a thing does or did, or what happened to a person or to a thing. The *Object* is the person or a thing upon whom or upon which the action of the verb is carried out.

The initial node of the graph is the predicate of the sentence. The search for the predicate is performed in *V P*. The initial node can be found in the following subtrees *V B* (verb, base form), *V BD* (verb, past tense), *V BG* (verb, present participle or gerund), *V BN* (verb, past participle), *V BP* (verb, present tense, not 3rd person singular), *V BZ* (verb, present tense, 3rd person singular), and *MD* (verb, modal). Further, from all solutions returned by searching for the predicate, the initial node is the verb chunk that is closest to the root of the sentence (number of edges from the verb node to the root node) and it is located in the verbal phrase that is closest to the root. The extracted predicate is stored in an entity called *Action*. We need to note here that because of the segmentation defined in the pre-processing step, which is done in order to extract all relevant information, it can happen that some sentence segments do not have a *V P*, so the *Action* entity is not returned.

If the *Action* entity is selected, all other entities of interest need to be selected. Because it can happen that no entity mention is the subject in the sentence, one additional entity called *Group* is added, into which the noun chunks that perform the action are stored. The *Group* entity is searched in the predecessor chunks from the *Action* entity that is selected. The search starts from the *Action* entity and then goes back to the beginning of the sentence. The results are the successive noun chunks that can also be separated by punctuation.

In order to know on which side of the *Action* entity the extracted entities are located, one of the labels *S*, *P*, or *O* that indicate (*Subject*, *Predicate*, *Object*) is added to each extracted entity. The *Action* entity has the label *P* because it is the predicate of the sentence. All entities that are predecessor chunks of the *Action* entity have the label *S* as they are subjects in the sentence. The entities that are successor chunks of the *Action* entity have the label *O* because they correspond to the objects in the sentence.

Two scenarios of entities selection exist. In the first one if the *Action* entity is not selected, then all the entities mentions from the *X*_Entities_ matrix are extracted. In the second scenario, only one *Action* entity is returned. Then for each entity using the set of its entities mentions, the entity mention or the chunk that is closest to the *Action* entity is selected, according to the number of edges between the candidate and the *Action* entity in the graph. If the set of entities mentions consists of more candidates for the same entity, they are extracted if they are on the same side from the *Action* entity as the one extracted or they are on the other side of the *Action* entity, but there is no additional verbal phrase in this part of the sentence.

## Results and discussion

In this section, we present the result of evaluation of the proposed NER method in the domain of evidence-based dietary recommendations. The main basic entities in the domain are the *Food* entity, *Nutrient* entity, and *Quantity*/*Unit* entity. For a better understanding of how the method works, we provide two examples. The first example focuses only on one sentence and the second one provides the results obtained from several sentences in order to present the concepts in which we are interested. Further, the construction of the heterogeneous test corpora that consists of text paragraphs is explained. Then, the obtained result using the explained test corpora is presented. Finally, we compare the methodology of the drNER with the methodologies of some other approaches that exist for biomedical domains.

### Examples

#### Example 1

To demonstrate how drNER works, we give an example where the focus is only on one sentence that provides dietary information.

Let Φ_1_ be the dietary recommendation “*People of any age who are African Americans should further reduce sodium intake to 300 mg per day.*” [16].

Table 6 gives results from the first phase of the NER method for Φ_1_. The *Tokens* column corresponds to the result of the word-level tokenization. The *POS* *tags* column corresponds to the result of the POS tagging. The *Chunk* *tokens* column corresponds to the result of the default chunking, where each chunk token is presented in the B-I-O tagging format and the beginning of each new chunk is marked with an *. The *Food*, *Nutrient*, and *Quantity*/*Unit* columns correspond to the linking of each token to the dictionaries used for each entity. In the column *Chunk*_1_ *tokens* the result of the first post-hoc chunking is presented, where new chunks formed by this chunking are presented in bold font. The new chunks formed by the second post-hoc chunking are presented in bold font in the *Chunk*_2_ *tokens* column. In the *Chunk*_3_ *tokens* column the result of the third post-hoc chunking is presented and in this example nothing is changed by applying this chunking.

**Table 6:** The first phase of the drNER method for Φ_1_.

Tokens	POS tags	Chunk tokens	Food	Nutrient	Quantity/Unit	Chunk_1_ tokens	Chunk_2_ tokens	Chunk_3_ tokens
People	NNS	B-NP*	0	0	0	B-NP*	B-NP*	B-NP*
of	IN	B-PP*	0	0	0	**I-NP**	I-NP	I-NP
any	DT	B-NP*	0	0	0	**I-NP**	I-NP	I-NP
age	NN	I-NP	0	0	0	**I-NP**	I-NP	I-NP
who	WP	B-NP*	0	0	0	B-NP*	B-NP*	B-NP*
are	VBP	B-VP*	0	0	0	B-VP*	**I-NP**	I-NP
African	JJ	B-NP*	0	0	0	B-NP*	**I-NP**	I-NP
Americans	NNP	I-NP	0	0	0	I-NP	**I-NP**	I-NP
should	MD	B-VP*	0	0	0	B-VP*	B-VP*	B-VP*
further	RBR	I-VP	0	0	0	I-VP	I-VP	I-VP
reduce	VB	I-VP	0	0	0	I-VP	I-VP	I-VP
sodium	NN	B-NP*	0	**1**	0	B-NP*	B-NP*	B-NP*
intake	NN	I-NP	0	0	0	I-NP	I-NP	I-NP
to	TO	B-PP*	0	0	0	B-PP*	B-PP*	B-PP*
300	CD	B-NP*	0	0	0	B-NP*	B-NP*	B-NP*
mg	NN	I-NP	0	0	**1**	**I-NP**	I-NP	I-NP
per	IN	B-PP*	0	0	0	**I-NP**	I-NP	I-NP
day	NN	B-NP*	0	0	0	**I-NP**	I-NP	I-NP
.	.	O	0	0	0	O	O	O

^1^ indicates the result of the first post-hoc chunking

^2^ indicates the result of the second post-hoc chunking

^3^ indicates the result of the third post-hoc chunking

* indicates a beginning of new chunk

Then, by using the Eq (3), the *X*_Entities_ matrix is calculated, where rows correspond to the different chunks and columns correspond to the entities, *Food*, *Nutrient*, and *Quantity*/*Unit*. The *X*_Entities_ matrix has 6 rows (different chunks) and 3 columns. The *Food* column gives the set of the entities mentions for the *Food* entity, which in our example is an empty set because the dietary recommendation does not consist of food entities. The *Nutrient* column gives the set of entities mentions for the *Nutrient* entity, and is a set with one element that is “sodium intake” identified by the row and the chunk that has a nonzero element in the *Nutrient* column. The *Quantity*/*Unit* column gives the set of the entities mentions for the *Quantity*/*Unit* entity, and is a set with one element that is “300 mg per day”.

**Fig 3:**
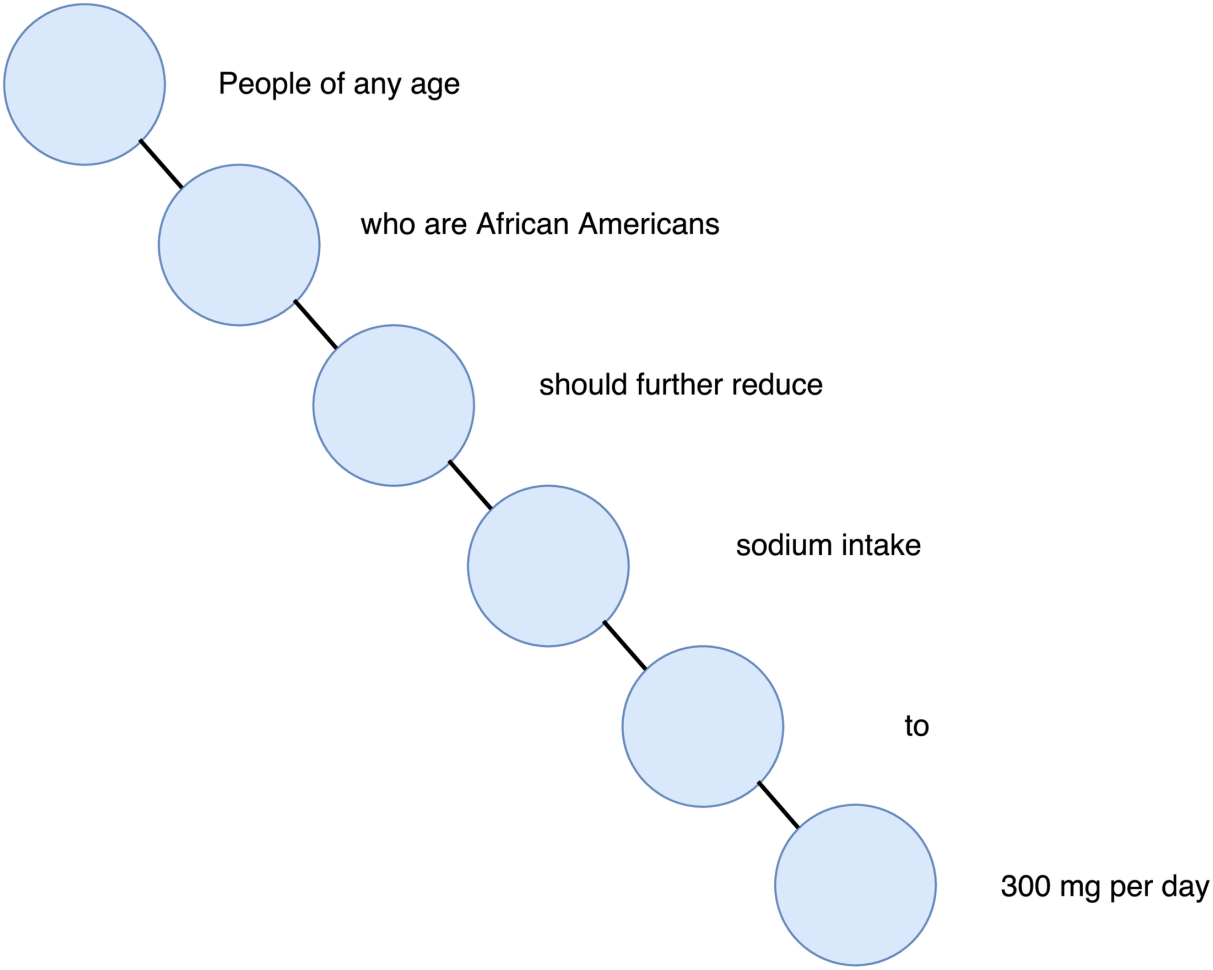
Graphic representation of the recommendation Φ_1_.

After the first phase, the recommendation Φ_1_ is represented as undirected graph, where each chunk is connected only with its neighbors. In Fig 3 a graphic representation of the recommendation Φ_1_ is presented.

**Fig 4:**
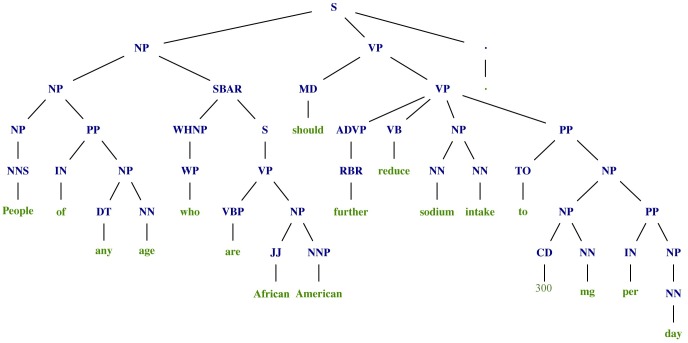
Parse tree of Φ_1_.

The first step of the second phase is to select the initial node of the graph or the *Action* entity from where the search for all entities will start. To select the *Action* entity the parse tree of the recommendation Φ_1_ is used. In Fig 4 we present the parse tree of the recommendation Φ_1_, from which we search for the predicate in the verbal phrases. The result is the verb “should” from the *MD* subtree, since it is closest to the root of the sentence. So, the chunk that consists of the returned verb, “should further reduce”, is selected as the *Action* entity.

The last step of the second phase is to select all other important entities. By using the second scenario (since the *Action* entity is returned), we found one *Nutrient* entity “sodium intake”, one *Quantity*/*Unit* entity “300 mg per day”, and for the *Group* entity we obtained “People of any age” and “who are African Americans”, while we did not find *Food* entity because there are no food related terms in the recommendation. At the end, the labels for the *Subject*, *Predicate* and *Object* are added (“People of any age”, *S*_1_), (“who are African Americans”, *S*_1_), (“should further reduce”, *P*_1_), (“sodium intake”, *O*_1_), and (“300 mg per day”, *O*_1_), which is also the result from our method. The index of the labels indicates from which part of the sentence the entity is extracted. In this example it is 1, because the recommendation does not contain any *ADV P*, *CONJP*, *SBAR*, or two successive chunks that are (*O*,*O*), so it is not split at the beginning.

#### Example 2

In the second example we present the results obtained for 15 sentences that are extracted from the Food and Nutrition Information Centre of United States Department of Agriculture [16]. This example helps the readers to get more familiar with the concepts from the dietary domain that need to be extracted. The results are presented in Table 7.

**Table 7:** Knowledge extraction of 15 dietary recommendations.

	Recommendation	Group	Action	Food	Nutrientt	Quantity/Unit
1.	Good sources of magnesium are: fruits or vegetables, nuts, peas and beans, soy products, whole grains and milk.	-	are (*P*_1_)	fruits or vegetables, nuts, peas and beans (*O*_1_) soy products (*O*_1_) whole grains and milk (*O*_1_)	Good sources of magnesium (*S*_1_)	-
2.	The RDAs for Mg are 300 mg for young women and 350 mg for young men.	-	are (*P*_1_)	-	The RDAs for Mg (*S*_1_)	300 mg for young women (*O*_1_) 350 mg for young men (*O*_1_)
3.	Increase potassium by ordering a salad, extra steamed or roasted vegetables, bean-based dishes fruit salads, and low-fat milk instead of soda.	-	-	salad (*S*_1_) extra steamed or roasted vegetables (*S*_1_) fruit salads (*S*_1_) law-fat milk (*S*_2_)	Increase potassium (*S*_1_) soda (*S*_3_)	-
4.	Babies need protein about 10 g a day.	Babies (*S*_1_)	need (*P*_1_)	-	protein (*O*_1_)	10 g a day (*O*_1_)
5.	1 teaspoon of table salt contains 2300 mg of sodium.	-	contains (*P*_1_)	table salt (*S*_1_)	sodium (*O*_1_)	1 teaspoon (*S*_1_) 2300 mg (*O*_1_)
6.	Milk, cheese, yogurt and other dairy products are good sources of calcium and protein, plus many other vitamins and minerals.	-	are (*P*_1_)	Milk, cheese, yogurt and other dietary products (*S*_1_)	good sources of calcium and protein (*O*_1_) many other vitamins and minerals (*S*_2_)	-
7.	Breast milk provides sufficient zinc, 2 mg/day for the first 4-6 months of life.	-	provides (*P*_1_)	Breast milk (*S*_1_)	sufficient zinc (*O*_1_)	2 mg/day for the first 4-6 months of life (*O*_1_)
8.	If you’re trying to get more omega-3, you might choose salmon, tuna, or eggs enriched with omega-3.	you (*S*_2_)	’re trying to get (*P*_2_) enriched (*P*_3_)	salmon, tuna, (*O*_2_) eggs (*S*_3_)	more omega-3 (*O*_2_) omega-3 (*O*_3_)	-
9.	If you need to get more fiber, look to beans, vegetables, nuts and legumes.	You (*S*_2_)	need to get (*P*_2_)	beans, vegetables, nuts, and legumes (*O*_2_)	more fiber (*O*_2_)	-
10.	Eating foods high in vitamin C and iron can reduce the absorption of ingested nickel.	-	can reduce (*P*_1_)	Eating foods (*S*_1_)	vitamin C and iron(*S*_1_) the absorption of ingested nickel (*O*_1_)	-
11.	The body of a 76 kg man contains about 12 kg of protein.	-	contains (*P*_1_)	-	protein (*O*_1_)	The body of a 76 kg man (*S*_1_) about 12 kg (*O*_1_)
12.	Excellent sources of alpha-linolenic acid, ALA, include flaxseeds and walnuts.	-	include (*P*_1_)	flaxseeds and walnuts (*O*_1_)	Excellent sources of alpha- linolenic acid (*S*_1_) ALA(*S*_1_)	-
13.	The recommended intake for total fiber for adults 50 years and younger is set at 38 g for men and 25 g for women, while for men and women over 50 it is 30 g and 21 g per day, respectively, due to decreased food consumption.	50 years (*S*_1_) younger (*S*_1_) for men and women over 50 (*S*_2_) it (*S*_2_)	is set (*P*_1_) is (*P*_2_)	decreased food consumption(*S*_3_)	The recommended intake for total fiber for adults (*S*_1_)	38 g for men (*O*_1_) 25 g for women (*O*_1_) 30 g (*O*_2_) 21 g per day (*O*_2_)
14.	I’m good at tennis.	-	-	-	-	-
15.	Your hat looks very nice.	-	-	-	-	-

If we look at sentence 5, “*1 teaspoon of table salt contains 2300 mg of sodium.*”, the recommendation is not split by the splitting proposed in the pre-processing part because it does not consist of *ADV P*, *CONJP*, *SBAR*, or two successive chunks that are (*O*,*O*). By using the drNER method (“table salt”, *S*_1_) is the *Food* entity extracted. There is one *Action* entity, (“contains”, *P*_1_), one *Nutrient* entity, (“sodium”, *O*_1_), and two *Quantity*/*Unit* entities, (“1 teaspoon”, *S*_1_) and (“2300 mg”, *O*_1_). The labels that are given to each of the extracted entities are the labels for the *Subject*, *Predicate*, and *Object* that help us better to interpret the extracted information. For example, in this dietary recommendation we have two *Quantity*/*Unit* entities, one of them is related to an entity extracted in the subject of the sentence, and the other one is related to an entity that is extracted in the object of the sentence. For example, from the label of the *Quantity*/*Unit* entity, (“1 teaspoon”, *S*_1_), we can see that this entity is related to some other entity that is extracted from the same part of the sentence, or the *Food* entity, (“table salt”, *S*_1_). The other *Quantity*/*Unit* entity, (“2300 mg”, *O*_1_), is related to an entity that is found in the object of the sentence, or in our case is related to the *Nutrient* entity, (“sodium”, *O*_1_). Finally, the extracted knowledge can be interpreted as ((“1 teaspoon”, *S*_1_), (“table salt”, *S*_1_)) and ((“2300 mg”, *O*_1_), (“sodium”, *O*_1_)), and (((“1 teaspoon”, *S*_1_), (“table salt”, *S*_1_)); (“contains”, *P*_1_); ((“2300 mg”, *O*_1_), (“sodium”, *O*_1_))).

Alternatively, the sentence 13, “*The recommended intake for total fiber for adults 50 years and younger is set at 38 g for men and 25 g for women, while for men and women over 50 it is 30 g and 21 g per day, respectively, due to decreased food consumption.*”, consists of two adverb chunks, so it needs to be split. If the recommendation is not split, than the entities “The recommended intake for adults” and “50 years” are extracted as *Group* entities, “is set” and “is” are extracted as *Action* entities, “decreased food consumption” is extracted as *Food* entity, and “38 g for men” and “25 g for women” are extracted as *Quantity*/*Unit* entities, and the information for men and women over 50 remains hidden, since it is not extracted. For this reason, the recommendation is split in the location of each adverb chunk. In this recommendation, there are two adverb chunks, “while” and “respectively”, so we split it in three parts, “*The recommended intake for total fiber for adults 50 years and younger is set at 38 g for men and 25 g for women.*”, “*For men and women over 50 it is 30 g and 21 g per day.*”, and “*Due to decreased food consumption.*”. The proposed method is then used on each part of the recommendation obtained after splitting. For the first part, the extracted entities are: (“The recommended intake for total fiber for adults”, *S*_1_) as *Nutrient* entity, (“50 years”, *S*_1_) and (“younger”, *S*_1_) as *Group* entities, (“is set”, *P*_1_) as *Action* entity, and (“38 g for men”, *O*_1_) and (“25 g for women”, *O*_1_) as *Quantity*/*Unit* entities. By applying the method on the second part of the recommendation, the extracted terms are (“For men and women over 50”, *S*_2_) and (“it”, *S*_2_) as *Group* entities, (“is”, *P*_2_) as *Action* entity, and (“30 g”, *O*_2_) and (“21 g per day”, *O*_2_) as *Quantity*/*Unit* entities. For the third part of the recommendation, only one extracted term exists (“decreased food consumption”, *S*_3_).

### Evaluation

#### Test corpora

**Fig 5:**
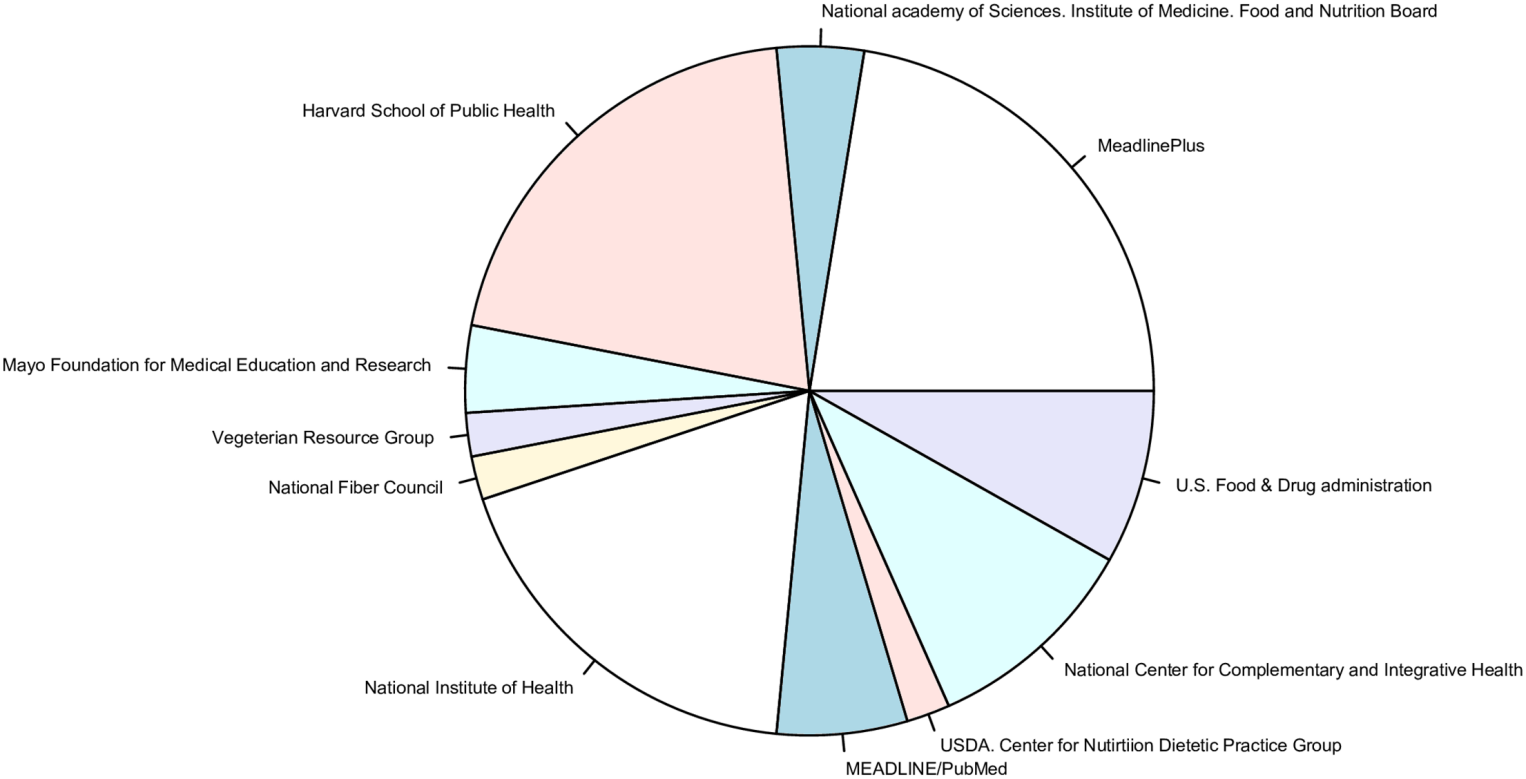
Distribution of documents per institution.

Due to the lack of annotated corpora in the domain of dietary information and in order to evaluate the newly proposed NER method for evidence-based dietary recommendations we created a test corpora. The main question was how to select the documents for the test corpora. We fixed the number at 100 because after extraction we need to manually check the extracted information. In order to promote diversity in the test corpora, we selected the documents from heterogeneous sources. We did this because different heterogeneous sources have different ways of reporting dietary recommendations. Fifty documents are dietary recommendation summaries, which are extracted from the scientifically validated web site, of the Food and Nutrition Information Center of United States Department of Agriculture [16]. These documents are extracted from 12 different institutions and their distribution per institution is presented in Fig 5.

**Fig 6:**
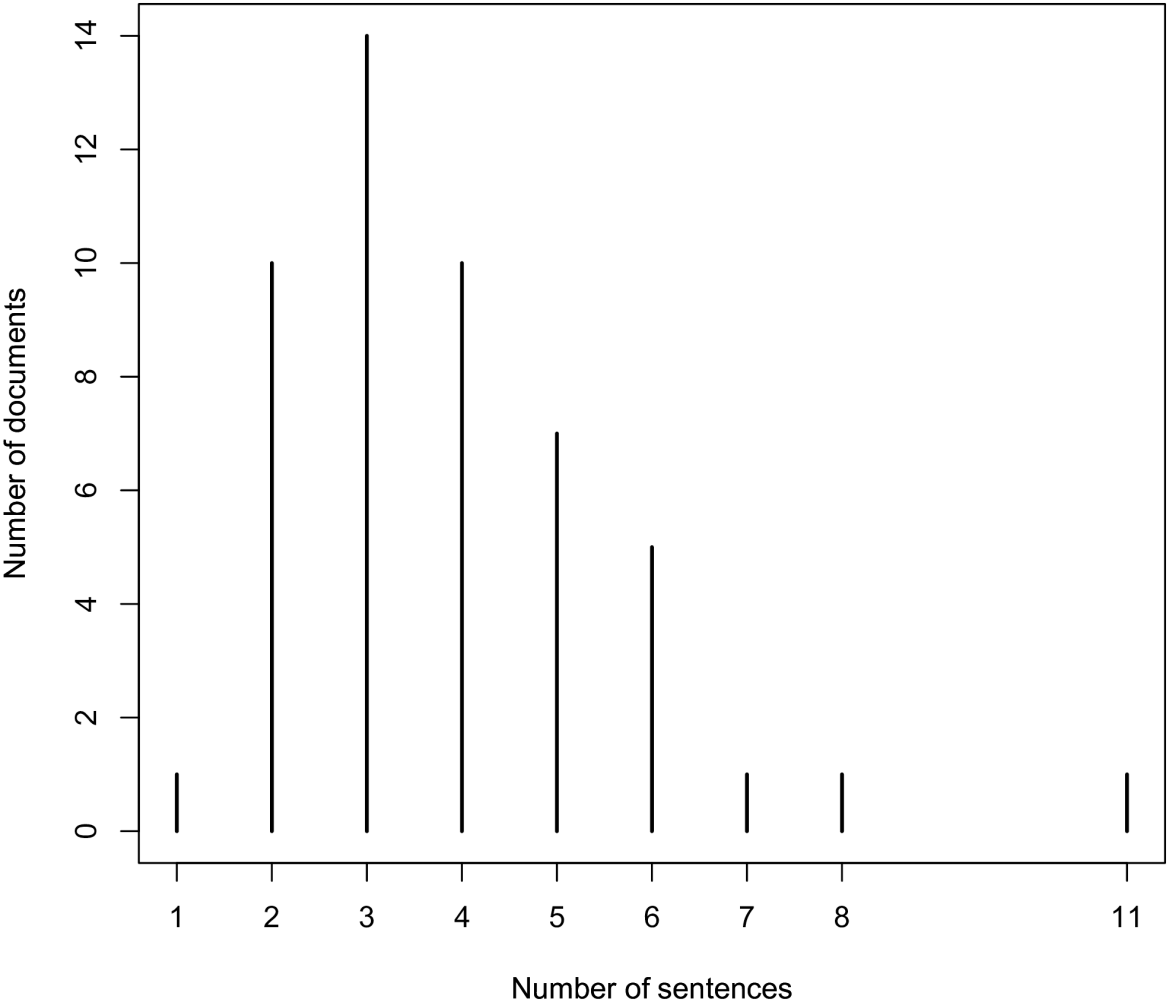
Distribution of number of sentences per documents from scientifically validated web sites.

**Fig 7:**
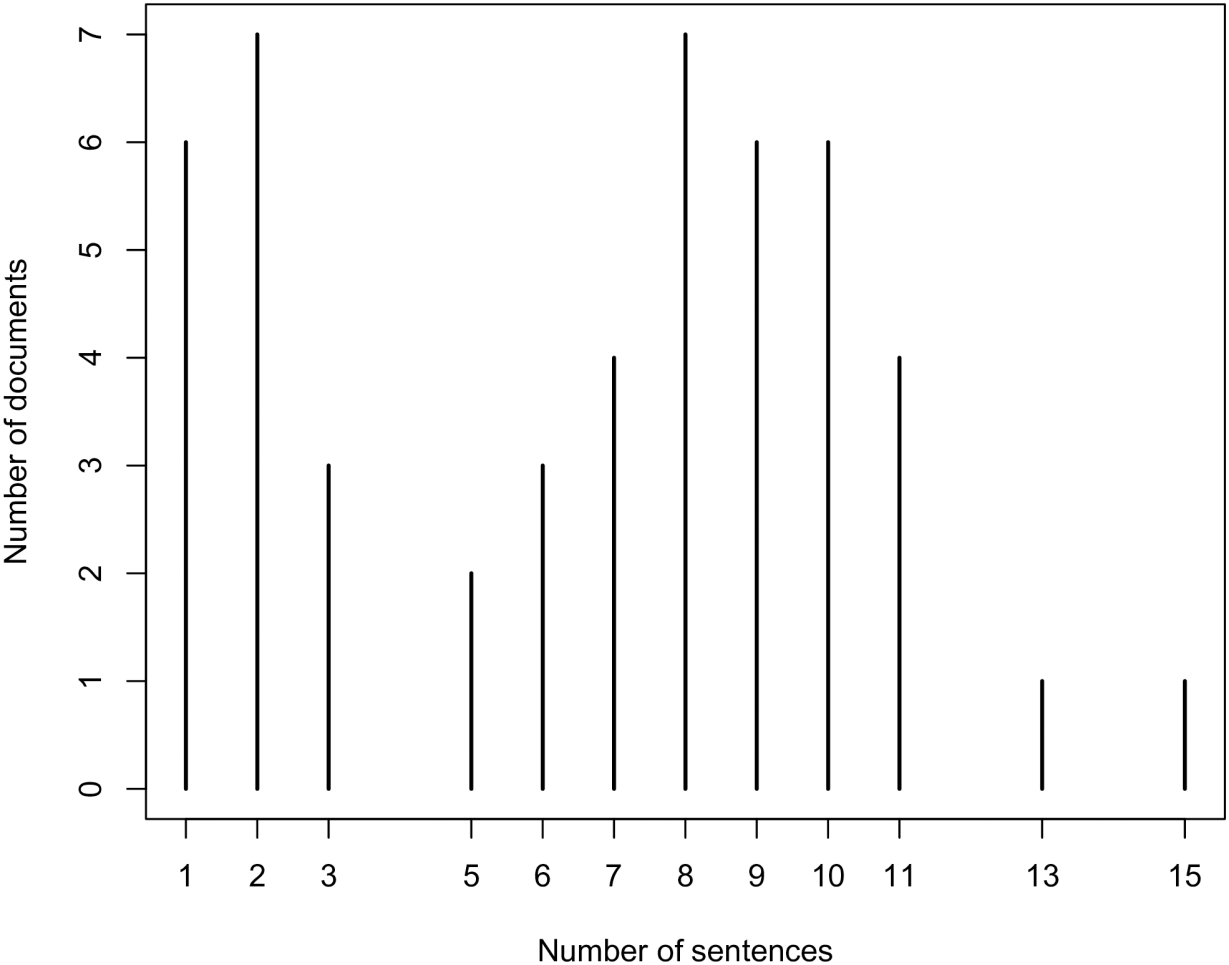
Distribution of number of sentences per documents that are abstracts from scientific publications.

The other 50 documents are abstracts of scientific publications. They were selected by using the PubMed API [85] in combination with two keywords, “food composition” and “dietary intake”. Further, 25 abstracts are selected randomly from the documents that are returned for each key word. The average number of sentences per document is 3.88 for documents from scientifically validated web sites and 6.54 for abstracts of scientific publications. This result is reasonable because the paragraphs from the scientifically validated web sites are summaries of dietary recommendations, while the abstracts from the scientific publications may contain dietary recommendations, but also contain other information about the study. The distribution of the number of sentences per document for the two subsets of the test corpora are presented in Figs 6 and 7.

This test corpora is not annotated. The results that are obtained by the drNER are further manually checked by 2 human experts, who are clinical dietitians, in order to see if the extracted entities have the correct label and if there are missing entities by drNER. The test corpora together with the obtained results for each document, separately, are available at the following link dx.doi.org/10.17504/protocols.io.hqbb5sn.

#### Result

We evaluated the drNER on the above test corpora. For each document in the test corpora, we tried to extract all useful information related to dietary recommendations, with focus on the *Food* entities, *Nutrient* entities, and *Quantity*/*Unit* entities. In Table 8, the result of the evaluation for each entity is presented. In this table, the results for the documents that are extracted from scientifically validated web sites and scientific publications are provided, separately. At the bottom of the table the summary of results of our test corpora is presented. The results are presented by reporting the number of true positives, false positives, and false negatives. The true positives are the extracted entities for which the obtained entity label from drNER and the human expert is the same. The false positives are the extracted entities for which the drNER label (known as expectation) is positive, but this is false according to the external judgment of the human expert. For example, for the *Food* label, if an extracted entity is labeled as *Food* by drNER, but the human expert provided that the true label is *Nutrient*, it means that this is false positive for the *Food* label. The false negatives are the entities for which the drNER label (known as expectation) is negative, but this is false according to the external judgment of the human expert. For example, for the *Food* label, if an extracted entity is not labeled as *Food* by drNER, but the human expert provided that the true label is *Food*, it means that this is false negative for the *Food* label. Also, in false negatives are some entities that are not recognized as a given entity of interest by drNER, but the human experts assumed that this information from the document should be extracted.

**Table 8:** Evaluation results.

	Food			Nutrient			Quantity/Unit		
	TP	FP	FN	TP	FP	FN	TP	FP	FN
Scientifically validated web sites	326	5	22	243	0	13	47	0	2
Scientific publications	213	0	3	314	2	4	39	0	9
Test corpora	539	5	25	557	2	17	86	0	11

*TP* indicates true positives

*FP* indicates false positives

*FN* indicates false negatives

The number of true positive *Food* entities is 539. Out of them 326 are from the documents that are extracted from scientifically validated web sites and 213 are from scientific publications. Also, there are 5 false positives, from the scientifically validated web sites, related to phrases that consist of verbs related to food such as “need to eat”, “eating”, etc., when they are not selected as an *Action* entity. In the future, this can be omitted by checking the tags of the chunks, so if they are verb chunks they can be removed from the candidate solutions. The number of false negatives for the *Food* entity is 25, out of which 22 are from the documents from the scientifically validated web sites and 3 of them are found in the scientific publications. They occur because “grains” is not recognized as a *Food* item by the USAS English semantic tagger we use as our dictionary. For some false negatives when the information about the *Food* entity is not extracted because it is not found in the dictionary, the information about it can be extracted in the *Group* entity that is proposed by the method to catch some additional useful information related to dietary information.

In the case of the *Nutrient* entity, the number of true positives in our test corpora is 557. Out of them, 243 are found from the documents extracted from the scientifically validated web sites and 314 are extracted from scientific publications. The number of false positives is 2, and the number of false negatives is 17. Out of them, 13 are extracted from the documents from scientifically validated web sites and 4 are found in the scientific publications. Most of them are related to the fact that none of the corpus-based NER, that we used as dictionaries, recognize the concepts as a chemical entity. Some of them are related to “omega-3s”. It is interesting that “omega-3” is recognized, but the plural form is problematic for all dictionaries we used. In the future, these results can be improved by adapting a heuristic approach for linking the tokens to the dictionaries.

For the *Quantity*/*Unit* entity, the number of true positives is 86. Out of them, 47 are from the documents extracted from the scientifically validated web sites and 39 of them are found in the scientific publications. We did not find any false positives and the number of false negatives is 11. The false negatives are related to some units such as “ngg(-1)”, “mgkg”, etc. This happens because these units do not exist in the dictionaries we used. Also, we use lemma of each token when we link it to the dictionaries related to the *Quantity*/*Unit* entity in order to distinguish between singular and plural forms of the units. We did this only for the *Quantity*/*Unit* entity because for others we used corpus-based NER systems that already include this information.

### Discussion

To the best of our knowledge, drNER is the first NER method that is focused on knowledge extraction of evidence-based dietary recommendations. The dietary domain brings a new application domain, with similar goals as previous IE shared tasks on biological event extraction. However, an annotated corpora does not exist, so there are no methods that focus on knowledge extraction. Because of that, a comparison of the drNER is made with some NER methods that can be used for each entity, separately, or they are NER methods that can in our case be used as dictionaries for some entities. For example, let us focus on one sentence “*People of any age who are African Americans should further reduce sodium intake to 300 mg per day.*” [16]. By using the USAS English online tagger and becas[chemicals] API, the *Nutrient* entity which will be extracted is “sodium”, while by using the becas API it is not recognized. By using the dictionaries applied for the *Quantity*/*Unit* entity, the only entity extracted here will be “mg”. The result by applying the drNER on this sentence is (“People of any age”, *S*_1_) and (“who are African Americans”, *S*_1_) as *Group* enitites, (“should further reduce”, *P*_1_) as *Action* entity, (“sodium intake”, *O*_1_) as *Nutrient* entity, and (“300 mg per day”, *O*_1_) as *Quantity*/*Unit* entity. If the recommendation is “*The RDAs for Mg are 300 mg for young women and 350 mg for young men.*”, by using the USAS English online tagger the result for the *Nutrient* entity is “*Mg*”, while by using the drNER it is “*The RDAs for Mg*”. So instead of extracting only the nutrient component, the drNER also could extract the type of the DRVs reported. In our proposed method, by applying the three proposed post-hoc chunkings, we can obtain also the phrases that differ from the phrases that can be obtained by the corpus-based NERs used as dictionaries and give us more information for the entities. Also, adding the *Action* entity and the labels for the *Subject*, *Predicate*, and *Object* provides additional information, and the *Group* entity helps to catch information that could be important to better interpret the extracted information.

To compare the methodology used in the drNER, we compare it with methodologies used by other NER methods used for other biomedical domains. For example, the SeeDev task that was a part of the 4th BioNLP Shared task consists two subtasks, SeeDev-binary on binary relation extraction and SeeDev-full on full event extraction. Because there is an annotated corpora, all 7 teams used supervised ML approaches. Five systems used SVMs and two systems were based on different algorithms, maximum entropy (MaxEnt) and a convolutional neural network. The methodology of the drNER method is completely different from the methodologies used by these approaches. The drNER is pure NLP method that is not based on annotated corpora, while all the SeeDev methods are based on ML approaches. Also, the entities involved in drNER are related to the dietary domain, and the SeeDev approaches involved in the extraction are related to genetic and molecular mechanisms involved in plant seed development. Another method is BANNER, evaluated on the BioCreative 2 GM training corpora, which is designed to maximize domain independence by not employing semantic features or rule-based processing steps. The domain-specific performance is not the purpose of the system, but researchers could adopt BANNER for a specific domain, by applying two types of post-processing. BANNER is based on annotated corpora and it uses CRFs. So the difference between the methodology used by BANNER and drNER is the same as the difference in the approaches presented on SeeDev task. Because an annotated corpora in the dietary domain does not exist, it is better to compare the methodology of the drNER with the methodologies of rule-based NERs that exist in the biomedical domains. Many rule-based NER methods use rules that combine terminological resources and the characteristics of the entities, but to write rules that depend from the characteristics for each entity is a time-consuming task. Further more, it requires a good understanding of the domain. For example, Lowe et al. [86] give a grammar and dictionary driven approach to entity recognition that uses a mixture of expertly curated grammars and dictionaries, as well as dictionaries automatically derived from public resources. They have created 486 rules. The benefit of this approach is that it works well when you do not have annotated data but requires dictionaries and grammars related to each entity. In our case, we do not have expertly created grammars for the entities we are interested in, but we only used a small number of Boolean algebra rules that are unrelated to the characteristics of the entities, but help us define the phrases that are entities mentions.

## Conclusion

In this paper we present a NER method for knowledge extraction of evidence-based dietary recommendations, called drNER. The goal of this method is to promote progress in information extraction in the field of dietary domain, especially focused on three main entities: *Food*, *Nutrient*, and *Quantity*/*Unit*. The dietary domain brings a new application domain, which has similar tasks on biomedical extraction, and is crucial for promoting health and well-being.

The proposed NER method for knowledge extraction of evidence-based dietary recommendations is a combination of terminological-driven NER and rule-based NER. The difference with the purely terminological-driven NER methods is that we allow for the use of corpus-based NERs as dictionaries for some entities of interest, instead of using dictionaries that consist of concepts and synonyms. The difference with the rule-based NERs is that we do not use rules based on the characteristics of the entities. We only have a small number of Boolean algebra rules that are not related to the characteristics, but help us to define the phrases that are entity mentions. The method consists of two-phases. The first phase involves the detection and determination of the entity mentions. It works by using some NLP methods and linking each token to a dictionary for each entity in which we are interested. After that, it uses three post-hoc chunkings in order to better determinate the entities mentions. The second phase is the selection and extraction of the entities. It is based on text syntactic analysis. Finally, by applying the rules defined in this phase, we can extract useful information related to dietary recommendations.

To the best of our knowledge, drNER is the first NER method where the focus is in the domain of evidence-based dietary recommendations, which is an untapped domain. The evaluation of drNER is done on test corpora that includes 100 documents. We fixed this number at 100 because an annotated corpora in this domain does not exist and after extraction, the extracted entities was manually checked by human experts to see if they have the correct labels. The test corpora included 50 summary paragraphs of dietary recommendations extracted from 12 different scientifically validated web sites and 50 abstracts of scientific publications that are related to “food-composition” and “dietary intake”. The best results achieved rely on the fact that for some entities such as *Food* and *Nutrient*, the terminological resources are not classic dictionaries that consist of concepts with synonyms, but they could be some corpus-based NERs that exist. For example, the *Nutrient* entity is related to chemical-named entity recognition. By using chemical NERs we can obtain the chemical information, but the type of DRVs or some additional information associated with it, is not extracted. For this purpose, three post-hoc chunkings are presented and help in modelling the dietary domain.

For future work, we plan to normalize the extracted entities. Then, we will try to find a good way to represent the extracted knowledge to human experts. By using the extracted knowledge from the dietary domain and the knowledge for drugs, diseases, and genes, that can be obtained from methods presented as a part of shared workshops, we will try to build an annotated corpora and to increase adoption of linked data techniques as an effective solution to knowledge representation and management in Life and Health Sciences [87]. Having an annotated corpora and knowledge representation, the next step will be to extract the relations that exists between these entities.
